# Prediction consistency and clinical presentations of breast cancer molecular subtypes for Han Chinese population

**DOI:** 10.1186/1479-5876-10-S1-S10

**Published:** 2012-09-19

**Authors:** Chi-Cheng Huang, Shih-Hsin Tu, Heng-Hui Lien, Jaan-Yeh Jeng, Jung-Sen Liu, Ching-Shui Huang, Yih-Yiing Wu, Chih-Yi Liu, Liang-Chuan Lai, Eric Y Chuang

**Affiliations:** 1 Graduate Institute of Biomedical Electronics and Bioinformatics, National Taiwan University, Taipei City, Taiwan; 2 Department of Surgery, Cathay General Hospital SiJhih, New Taipei City, Taiwan; 3 School of Medicine, Fu-Jen Catholic University, New Taipei City, Taiwan; 4 School of Medicine, Taipei Medical University, Taipei City, Taiwan; 5 Department of Surgery, Cathay General Hospital, Taipei City, Taiwan; 6 Department of Pathology, Cathay General Hospital SiJhih, New Taipei City, Taiwan; 7 Graduate Institute of Physiology, National Taiwan University, Taipei City, Taiwan

## Abstract

**Background:**

Breast cancer is a heterogeneous disease in terms of transcriptional aberrations; moreover, microarray gene expression profiles had defined 5 molecular subtypes based on certain intrinsic genes. This study aimed to evaluate the prediction consistency of breast cancer molecular subtypes from 3 distinct intrinsic gene sets (Sørlie 500, Hu 306 and PAM50) as well as clinical presentations of each molecualr subtype in Han Chinese population.

**Methods:**

In all, 169 breast cancer samples (44 from Taiwan and 125 from China) of Han Chinese population were gathered, and the gene expression features corresponding to 3 distinct intrinsic gene sets (Sørlie 500, Hu 306 and PAM50) were retrieved for molecular subtype prediction.

**Results:**

For Sørlie 500 and Hu 306 intrinsic gene set, mean-centring of genes and distance-weighted discrimination (DWD) remarkably reduced the number of unclassified cases. Regarding pairwise agreement, the highest predictive consistency was found between Hu 306 and PAM50. In all, 150 and 126 samples were assigned into identical subtypes by both Hu 306 and PAM50 genes, under mean-centring and DWD. Luminal B tended to show a higher nuclear grade and have more HER2 over-expression status than luminal A did. No basal-like breast tumours were ER positive, and most HER2-enriched breast tumours showed HER2 over-expression, whereas, only two-thirds of ER negativity/HER2 over-expression tumros were predicted as HER2-enriched molecular subtype. For 44 Taiwanese breast cancers with survival data, a better prognosis of luminal A than luminal B subtype in ER-postive breast cancers and a better prognosis of basal-like than HER2-enriched subtype in ER-negative breast cancers was observed.

**Conclusions:**

We suggest that the intrinsic signature Hu 306 or PAM50 be used for breast cancers in the Han Chinese population during molecular subtyping. For the prognostic value and decision making based on intrinsic subtypes, further prospective study with longer survival data is needed.

## Background

In the past decade, microarray experiments have redefined breast cancers as heterogeneous diseases in terms of transcriptional aberrations, and a number of taxonomic classifications based on gene-expression profiles that have been reported have shown some prognostic significance. One such molecular taxonomy is the ‘intrinsic subtype’ proposed by the Stanford group. Perou identified 476 intrinsic genes from 65 patients with breast cancers and normal individuals; four subclasses: basal-like, Erb-B2+, normal breast-like, and luminal epithelial/ER+ were revealed by class discovery through clustering analysis [1,2]. The luminal subtype was further divided into luminal A and B, and distant metastases were strongly associated with the expression patterns of intrinsic genes [3]. Independent studies supporting the existence of breast cancer intrinsic subtypes followed [4,5]. By definition, intrinsic genes were those genes that show the highest variation across different subjects and show the least variation within each individual (i.e. pre-/post-chemotherapy changes) [3]. The latest version of intrinsic signatures, prediction analysis of microarray 50 gene set (PAM50), was supposed to provide prognostic and predictive values independent of traditional prognostic factors such as hormone receptor, human epidermal growth factor receptor 2 (HER2) over-expression, or proliferation markers [6].

Although much attention has been drawn and intense arguments have been made, serious concerns about the true existence and reproducibility of intrinsic signatures remain. For instance, Lusa debated the comparability of study populations and concluded that assigning of new samples, which were not a part of the original dataset from which the intrinsic genes were derived to molecular subtypes was elusive [7]. Recently, Weigelt compared the agreement in subtype assignment across 3 different intrinsic genes-based single sample predictor (SSP) and found only fair to substantial pairwise agreement [8]. Indeed, the degree of overlap between distinct intrinsic gene lists was surprisingly low, although most of these molecular signatures were claimed to have some prognostic values [9]. The reproducibility and robustness of molecular subtypes from intrinsic genes by hierarchical clustering was also challenged [7,10].

In the current study, we evaluated the application of molecular subtypes across ethnic groups. Predictive consistency across different intrinsic gene sets as well as the impact of systemic microarray bias adjustment was assessed for Taiwanese and Chinese breast cancer patients, both of Han Chinese origin. Clinical and pathological features of each molecular subtype were compared accordingly.

## Methods

### Study population and microarray experiments

The study material included 169 breast cancers of Han Chinese population; 44 were from Taiwan, and 125, from Mainland China. Regarding the Taiwanese samples, sporadic breast cancer samples were collected consecutively during surgery, snapped frozen in liquid nitrogen, and then stored and transported at -80^0^C from January 2007 to January 2008. The frozen samples were dissected into slices of 1-2 mm thickness, and more than 90% of the cancerous content was a pre-requisite for microarray experiments. All examinations and management of the surgical specimens were carried out by 2 qualified pathologists (YYW and CYL). Written consent was obtained for all subjects before sample collection, and the study protocol was approved by Institute Review Board of Cathay General Hospital. The criteria of enrolment included incident/invasive breast cancers without neo-adjuvant therapy, no systemic spread (clinical stage I to III), no concurrent secondary malignancy, and less than 70 years of age.

Total RNA was extracted by TRIzol® reagent (Invitrogen, Carlsbad, CA) and the RNA was purified using RNeasy® mini kits (Qiagen, Germantown, MD). RNA integration was tested by gel electrophoresis. Affymetrix® (Affymetrix, Santa Clara, CA) GeneChip® Human Genome U133 plus 2.0 was used for the microarray experiment. Hybridization and scanning were performed according to a standard protocol. Images were scanned using GeneChip® Scanner 3000, and the scanned images were processed with GeneChip® Operating Software (GCOS). Robust multi-array average (RMA) algorithm was used to normalize 44 array chips [11]. For the 125 samples from China, raw expression files (CEL files) were downloaded from NCBI Gene Expression Omnibus (GSE5460) by using the same Affymetrix® U133 plus 2.0 arrays and normalized by RMA; details of the study are described elsewhere and the study was approved by local IRB [12]. The processed expression profiles of breast cancers from Taiwan and Mainland China were pooled together, and quantile normalization was performed to remove the batch effect between the breast cancer in Taiwanese patients and Chinese patients.

For relevant pathological features, estrogen receptor (ER) positivity was defined as the presence of at least 10% of nuclei with positive results of immunohistochemical (IHC) analysis, and breast samples displaying low ER positivity (1-9% of nuclei with positive stains) were not assayed in current study. For HER2 status, the ASCO and CAP guidelines were followed; IHC 3+ and IHC 2+ with fluorescence in-situ (FISH) hybridization amplification were considered to indicate HER2 over-expression. The modified Bloom-Richardson (Nottingham) system was used for grading breast cancers. The demographic features of the 169 Han Chinese patients with breast cancers are summarized in Table 1.

**Table 1:** Demographic features of study population

Source	Taiwan		China		Total
	n=44		n=125		n=169
ER					
Positive	22(50%)		74(59%)		96(57%)
Negative	22(50%)		51(41%)		73(43%)

HER2					
Over-expressed	21(48%)		30(24%)		51(30%)
Not	23(52%)		95(76%)		118(70%)

Nuclear grade					
I	3(7%)		27(22%)		30(18%)
II	18(41%)		31(25%)		49(29%)
III	23(52%)		67(54%)		90(53%)

Nodal status					
Positive	23(52%)		61(49%)		84(50%)
Negative	21(48%)		64(51%)		85(50%)

Lympovascular invasion*					
Positive	27(63%)		46(37%)		73(43%)
Negative	16(37%)		79(63%)		95(57%)

*one missing value

### Intrinsic gene lists and prototypical samples

Three intrinsic signatures that defined 5 molecular subtypes (luminal A, luminal B, normal breast-like, HER2-enriched, and basal-like) were Sørlie 500, Hu 306 and PAM50 [3,4,6]. These different sets of intrinsic genes presented a chronological evolution of molecular subtypes proposed by the Stanford group. The expression values of training samples deriving intrinsic signatures were downloaded from Stanford Genomics Breast Cancer Consortium and UNC Microarray Database (see Additional file 1 for details). Centroids were the mean expression values of intrinsic genes corresponding to each molecular subtype.

### Single sample prediction and systemic bias adjustment

All intrinsic genes were mapped to the Affymetrix gene annotation file, and the data of the genes represented by multiple-probesets were averaged (see Additional file 1 for details of mapping process). The 169 breast cancer specimens of the Han Chinese patients were assigned to 1 of the 5 molecular subtypes with the nearest centroid (single sample prediction). Spearman’s rank correlation coefficients were used, and samples were designated as unclassified if correlation coefficients to all 5 centroids were less than 0.1.

To enhance the comparability between the original studies deriving intrinsic genes and independent samples in current study, we applied 2 systemic bias-adjustment methods, mean-centring of genes and distance-weighted discrimination (DWD), to the expression data of Han Chinese breast cancers, as suggested by the investigators of the Stanford group [13,14].

## Results

### Distributions of molecular subtypes

Table 2 shows the distribution of molecular subtypes under different combinations of intrinsic genes and adjustment methods. Without adjustment, both Sørlie 500 and Hu 306 identified many unclassified samples, and the number of unclassified samples reduced rapidly when gene centring or DWD was applied, indicating the necessity of bias adjustment across microarray studies. The shift between the 2 luminal subtypes A and B was prominent with and without adjustment; however, the direction of this shift was unpredictable from the current study. On the other hand, PAM50 was less sensitive to systemic microarray bias correction. Regardless of the adjustment, the least changes were observed for the basal-like subtype across all 3 intrinsic genes. We also noticed that PAM50 identified many normal breast-like tumours under DWD.

**Table 2:** Molecular subtype distrubutions of 169 Han Chinese breast cancers with different intrinsic genes and adjustment

Intrinsic genes	Original data without adjustment	Mean-centring of genes	DWD adjustment
Sørlie 500			
Luminal A	36	69	70
Luminal B	60	24	23
Normal breast-like	4	11	15
Basal-like	37	37	44
HER2-enriched	2	21	13
Unclassified	30	7	4
			
Hu 306			
Luminal A	84	58	57
Luminal B	1	32	35
Normal breast-like	0	8	10
Basal-like	30	41	41
HER2-enriched	16	29	25
Unclassified	38	1	1
			
PAM50			
Luminal A	70	56	51
Luminal B	32	36	19
Normal breast-like	10	6	27
Basal-like	36	41	41
HER2-enriched	19	30	31
Unclassified	2	0	0

### Agreement between adjustment methods with the same intrinsic genes

For Sørlie 500 and Hu 306, mean-centring of genes and DWD showed good agreement in subtype assignment (unweighted kappa: 0.83 and 0.95, respectively). For PAM50, gene centring and original data showed the highest predictive consistency (unweighted kappa: 0.80), followed by gene centring and DWD, then original data and DWD (unweighted kappa: 0.67 and 0.66, respectively, Table 3).

**Table 3:** Agreement between intrinsic gene sets and adjustment methods

Intrinsic genes	Kappa*	95% CI	Adjustment method	Kappa*	95% CI
Sørlie 500			Gene centring		
Original data vs. gene centring	0.51	0.43-0.60	Sørlie 500 vs. Hu 306	0.58	0.50-0.67
Original data vs. DWD	0.49	0.41-0.57	Sørlie 500 vs. PAM50	0.52	0.43-0.61
Gene centring vs. DWD	0.83	0.77-0.90	Hu306 vs. PAM50	0.85	0.79-0.91

Hu 306			DWD adjusted		
Original data vs. gene centring	0.51	0.43-0.58	Sørlie 500 vs. Hu 306	0.56	0.47-0.65
Original data vs. DWD	0.5	0.42-0.58	Sørlie 500 vs. PAM50	0.55	0.47-0.64
Gene centring vs. DWD	0.95	0.92-0.99	Hu306 vs. PAM50	0.67	0.59-0.76

PAM50					
Original data vs. gene centring	0.8	0.73-0.87			
Original data vs. DWD	0.66	0.58-0.75			
Gene centring vs. DWD	0.67	0.58-0.75			

### Agreement between intrinsic gene sets with the same adjustment

Table 3 also shows pairwise agreement between the 3 intrinsic gene sets. When genes were mean-centred, Hu306 and PAM50 showed the highest agreement (unweighted kappa: 0.85), and the consistency dropped substantially (unweighted kappa: 0.67) when DWD was adopted for adjustment. Sørlie 500 intrinsic genes showed only substantial agreement with Hu 306 and PAM50 under gene centring or DWD (see Additional file 2 for supplementary Table S2).

### Clinical features of molecular subtypes

The breast cancer samples assigned to identical subtypes with both Hu 306 and PAM50 intrinsic genes were retrieved. These included samples from 150 (89%) and 126 (75%) of the 169 study subjects under gene centring and DWD adjustment, respectively. In the most stringent conditions, 117 (69%) samples were consistently assigned to identical subtypes under both DWD and gene centring. Their clinical features are presented in Table 4 (supplementary Table S1 for molecular subtypes stratified by clinical phenotypes); luminal B tended to show a higher nucler grade and HER2 over-expression than luminal A did. No basal-like breast tumours were ER positive, and most HER2-enriched breast tumours showed HER2 over-expression and were clinically ER negative, whereas, only two-thirds of tumours that showed ER negativity/HER2 over-expression were predicted as HER2-enriched molecular subtype.

**Table 4:** Clinical features of agreeing samples between Hu 306 and PAM50 (n=117 with both mean-centring and DWD adjustment)

Clinical factor	Molecular subtype				
	Luminal A	Luminal B	Normal-breast like	Basal-like	HER2-enriched
ER					
Positive	35	15	2	0	3
Negative	0	1	4	39	18
					
HER2					
over-expressed	2	5	2	2	18
not over-expressed	33	11	4	37	3
					
Nuclear grade					
I	17	0	1	0	0
II	16	4	2	2	4
III	2	12	3	37	17
					
Nodal status					
Positive	16	9	5	10	14
Negative	19	7	1	29	7
					
Lymphovascular invasion*					
Positive	14	11	4	11	13
Negative	21	5	2	28	8

*One missing value

**Figure 1:**
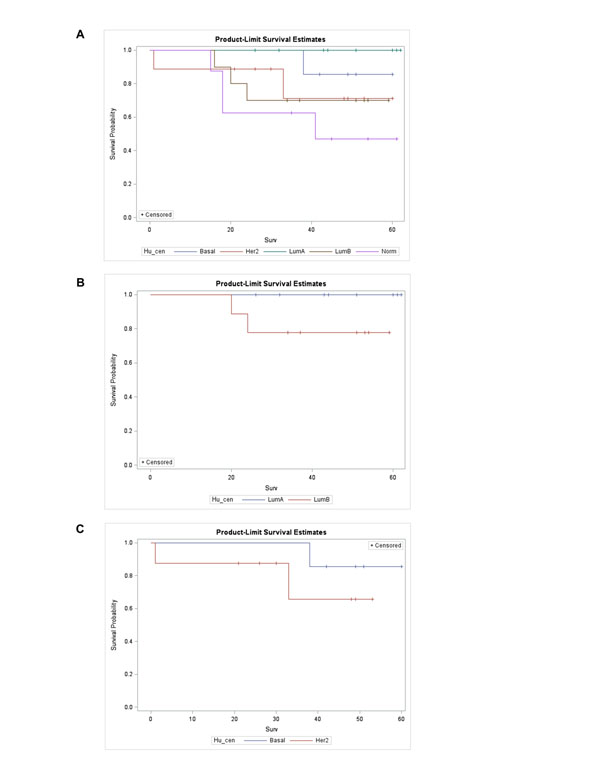
Survival analysis of 44 Taiwanese breast cancers with Hu 306 intrinsic genes and mean-centring adjustment (a). Subgroup analysis in ER-positive breast cancers (n=19), only luminal A and B subtypes displayed due to the sparseness of other molecular subtypes (b). Subgroup analysis in ER-negative breast cancers (n=15), only basal-like and HER2-enriched subtypes displayed due to the sparseness of other molecular subtypes (c). (abbreviations: LumA: luminal A, LumB: luminal B, Her2, HER2-enriched, basal: basal-like, Norm: normal breast-like subtype, Hu_cen: Hu 306 intrinsic genes with mean-centring adjustment).

For 44 Taiwanese breast cancers, clinical follow-up was available up to 62 months (median:44.5). There were 10 events of distant metastasis or mortality attributed to breast cancers. Figure 1(a) showed that there was no significant event-free survival difference among the 5 molecular subtypes (log-rank test: 0.13, with Hu 306 intrinsic genes and gene-centring adjustment), better prognosis of luminal A and compromised survival of luminal B and HER2-enriched subtype was apparent though. For ER-positive patients, luminal A outperformed luminal B while in ER-negative cancers, the prognosis of basal-like subtype was better than HER2-enriched subtype for Taiwanese breast cancers (Figure 1(b) and 1(c), respectively).

## Discussion

In the current study, we evaluated the trans-ethnic applicability of breast cancer molecular subtypes for independent samples obtained from people of the Han Chinese origin. Three distinct intrinsic gene sets were used, and the systemic microarray bias was accounted for by carrying out mean-centring of genes or DWD. We found that without adjustment, Sørlie 500 and Hu 306 signatures had a higher proportion of unclassified cases, thereby highlighting the importance of eliminating systemic bias and enhancing comparability across microarray studies. This was further supported by the high kappa statistics between DWD and gene centring when Sørlie 500 and Hu 306 intrinsic genes were used. Among all the 3 intrinsic gene sets, PAM50 was less sensitive to systemic adjustment but showed more normal breast-like samples (especially with DWD).

The number of breast cancer samples predicted as normal breast-like subtype was largely influenced by intrinsic genes used and adjustment method. Notably, normal breast-like centroid in PAM50 was derived from 29 normal breast samples and should be treated as an internal quality control rather than a breast cancer intrinsic variation in Hu 306 and Sørlie 500 [6]. In this sense, none of our samples should be predicted as normal breast-like subtype with PAM50. In our study, the higher number of samples catogorised as the normal breast-like subtype by PAM50 indicates possible normal breast tissue contamination without laser capture microdissection, or dubious predictive accuracy of PAM50 normal breast-like centroid derived from unrepresentative or limited number of normal breast samples. Another possible explanation is that gene expression values were obtained by microarray experiments rather than reserve transcriptase polymerase chain reaction (RT-PCR) used by the PAM50 signature, and this led to some discrepancies in measurements.

We next compared the pairwise consistency between the distinct intrinsic gene sets, under the same adjustment manner. With gene centring, Hu 306 and PAM50 showed the highest consistency (unweighted kappa: 0.85), or nearly 90% of the assayed samples were identified to be of the same subtype. Hu 306 and PAM50 still showed the highest agreement when adjusted by DWD, but only substantial agreement (unweighted kappa: 0.67) was observed. Because Sørlie 500 appeared the first in all 3 intrinsic signatures, many of its clone ID identifiers had difficulties mapping to the latest HUGO gene symbol and modern microarray platform used in current study. Investigators of the Stanford group also suggested the use of the most recently updated PAM50 during molecular subtyping of clinical samples [6,13]. Here, we discourage the use of Sørlie 500 gene set for intrinsic subtyping. Regarding adjustment method, gene-centring rather than DWD might deliver more optimisitc results since fewer normal breast-like samples were predicted, especially when PAM50 intrinsic genes were used.

Some researchers might tend to use clinical phenotypes as surrogates for breast cancer molecular subtypes such that ER-positive tumours were analogous to luminal subtype, tumours with ER-negativity/HER2 over-expression were analogous to HER2-enriched subtype, and tumours with ER-negativity/HER2 negativity were analogous to basal-like breast cancer [15,16]. Supplementary Table S3 (Additional file 3) showed the intrinsic subtype distrubutions stratified by so-called “IHC subtype” in our study and as expected, there was a substantial disagreement between molecular subtype defined by intrinsic genes and subtype determined by conventional IHC methods alone. However, molecular subtypes were determined to be independent of pathological markers and the investigtors of PAM50 also argued that ER and HER2 status alone were not accurate surrogates for ‘true’ intrinsic subtype status; for instance, only 64% of cases with HER2 over-expression were designated as HER2-enriched subtype in their study [6]. The discrepancy between clinical HER2 phenotype and molecular HER2-enriched subtype raised the serious concern that whether these patients should be managed according to IHC/FISH results or gene expression profiles and this dispute remained inconclusive. In table 4 we summarized pathological features of each molecular subtype from samples presistantly predicted into identical subtype by Hu 306 and PAM50 under both DWD and gene-centring adjustment (roughly 70% of all assayed samples). As expected, luminal B showed more aggressive behaviors by traditional prognostic features, all basal-like tumros were ER negative, most HER2-enriched tumors showed IHC/FISH HER2 overe-expression, whereas only two-thirds of tumours with clinical ER-negativity/HER2 over-expression were predicted as HER2-enriched subtype according to the gene expression assays, and these findings were grossly in concordance with our knowledge about each breast cancer molecular subtype. For 44 Taiwanese breast cancers with survival data, we did find a trend toward good prognosis for luminal A subtype and worse prognosis for luminal B and HER2-enriched subtype, and a survival benefit of basal-like over HER2-enriched subtype was also observed, especially when subgroup analysis was performed according by IHC ER status.

Our study had some limitations. First, we did not have a sufficient sample size to determine the prognostic value of each molecular sutbype upon breast cancer survival and multi-variate analysis incorporating clinical and pathological factors was not possible. Second, a prospective study design is needed to eliminate selection bias and may be help to incorporate breast cancer molecular subtypes into clinical decision makings.

## Conclusions

In the current study we evaluted the prediction consistency and clinical presentations of breast cancer molecular signatures trans-ethnically for Han Chinese population. We found that with proper adjustments to enhance comparability across microarray studies, the predictive consistency between PAM50 and Hu 306 was achieved for nearly 90% of our independent breast cancer samples, and disparities in the associated clinical and pathological features were observed between distinct molecular subtypes. A trend of prognostic disparity was also observed from intrinsic subtypes among Taiwanese breast cancers, and provides an opportunity for developing risk prediction models and dissecting the heterogeneity within ER positive and negative breast cancers respectively. Further work to evaluate the relevance of molecular subtypes and survival should be initiated using more clinical samples with longer follow-up of patients.

## Abbreviations

ER: estrogen receptor; HER2: human epidermal growth factor receptor 2; DWD: distance-weighted discrimination; SSP: single sample predictor; IHC: immunohistochemical; RT-PCR: reserve transcriptase polymerase chain reaction

## Competing interests

The authors declare that they have no competing interests.

## Authors' contributions

CCH initiated the study and drafted the manuscript. SHT, HHL, JYJ and CSH participated in the study design, sample collection and shared experts’ opinions. YYW and CYL handled the frozen samples and performed all pathological examinations. JSL provided statistical help and interpretation of results. LCL carried out microarray experiments. EYC conceived the whole study and took the responsibility of corresponding author. All authors read and approve the final manuscript.

## Supplementary Material

Additional file 1 **Supplementary materials and methods** This file included the mapping process of intrinsic genes to Affymetrix® probesets, and the source of Chinese breast cancer microarrays. Table S1 summarized intrinsic subtype distributions stratified by clinical phenotypes. Click here for file

Additional file 2 **Supplementary Table S2** Supplementary Table S2 contained pairwise comparisons between 3 intrinsic gene lists for the assignment of the samples. Click here for file

Additional file 3 **Supplementary Table S3** Table S3 showed the distributions of molecular subtypes defined by intrinsic genes (Hu 306 and PAM50 with gene-centring and DWD) stratified by subtypes defined by IHC results. Click here for file
