# Supplementary material for: Prediction consistency and clinical presentations of breast cancer molecular subtypes for Han Chinese population
Source: J Transl Med. 2012 Sep 19;10(Suppl 1):S10. doi: 10.1186/1479-5876-10-S1-S10 (PMC3445863; doi:10.1186/1479-5876-10-S1-S10)
Supplement: Additional file 1 — Supplementary materials and methods This file included the mapping process of intrinsic genes to Affymetrix® probesets, and the source of Chinese breast cancer microarrays. Table S1 summarized intrinsic subtype distributions stratified by clinical phenotypes. [file 1479-5876-10-S1-S10-S1.pdf]

Additional file 1 – Supplementary materials and methods

Sørlie 500 intrinsic genes

The Sørlie 500 intrinsic gene lists and prototypical arrays were obtained from Stanford Genomics Breast Cancer Consortium. 552 IMAGE Clone ID identifiers were mapped to the latest HUGO gene symbols, then to the Affymetrix gene annotation file. This resulted in 1098 Affymetrix probesets representing 461 genes. The prototypical arrays included 28 luminal A, 11 luminal B, 11 Her2-enriched, 19 basal-like and 10 normal breast-like tumors (all samples with correlation coefficients less than 0.1 were discarded). For DWD adjustment, missing values in training dataset were imputed using 10-NN method.

Hu 306 intrinsic genes

The Hu 306 intrinsic gene lists and prototypical arrays were obtained from UNC Microarray Database. 306 Agilent probeset identifiers were mapped to the latest HUGO gene symbols, then to the Affymetrix gene annotation file. This resulted in 783 Affymetrix probesets representing 300 genes. The prototypical arrays included 46 luminal B, 136 luminal A, 19 Her2-enriched, 65 basal-like and 29 normal breast-like tumors. For DWD adjustment, missing values in training dataset were imputed using 10-NN method.

PAM50 intrinsic genes

The PAM50 intrinsic genes and prototypes were downloaded from UNC Microarray Database. 50 RT-PCR gene names were mapped to 117 Affymetrix probesets. The prototypes included 12 normal breast-like, 57 basal-like, 35 Her2-enriched, 23 luminal A and 12 luminal B tumors. Missing values in training dataset were imputed using 10-NN method.

GSE 5460 dataset

The GSE5460 included 129 Affymetrix® U133 plus 2.0arrays and four microarray experiments (GSM125119, GSM125120, GSM125125, GSM125122) were not used in current study due to technical problems reading these CEL files.

Table S1 - Molecular subtype distributions of 169 Han Chinese breast cancers stratified by clinical phenotypes

Intrinsic genes		Sørlie 500			Hu 306			PAM50		
Adjustment		Original data	Gene centring	DWD	Original data	Gene centring	DWD	Original data	Gene centring	DWD
ER+Her2 n=73	Luminal A	33	60	61	69	55	53	57	51	43
	Luminal B	14	3	1	1	16	18	14	20	11
	Normal breast-like	2	7	11	0	2	2	2	2	19
	Basal-like	0	0	0	0	0	0	0	0	0
	Her2-enriched	0	0	0	0	0	0	0	0	0
	Unclassified	24	3	0	3	0	0	0	0	0
ER+Her2 n=23	Luminal A	2	7	7	9	3	4	8	3	6
	Luminal B	18	10	12	0	13	14	13	14	6
	Normal breast-like	0	0	0	0	0	0	0	0	0
	Basal-like	0	0	0	0	0	0	0	0	0
	Her2-enriched	0	2	2	2	6	4	2	6	11
	Unclassified	3	4	2	12	1	1	0	0	0
ER-Her2- n=45	Luminal A	1	2	2	4	0	0	0	0	1
	Luminal B	5	0	0	0	2	2	4	2	1
	Normal breast-like	1	2	2	0	2	2	4	2	2
	Basal-like	36	35	37	28	38	38	34	37	37
	Her2-enriched	0	6	3	0	3	3	2	4	4
	Unclassified	2	0	1	13	0	0	1	0	0
ER- n=28	Luminal A	0	0	0	2	0	0	5	2	1
	Luminal B	23	11	10	0	1	1	1	0	1
	Normal breast-like	1	2	2	0	4	6	4	2	6
	Basal-like	1	2	7	2	3	3	2	4	4
	Her2-enriched	2	13	8	14	20	18	15	20	16
	Unclassified	1	0	1	10	0	0	1	0	0
