# Supplementary material for: Prediction consistency and clinical presentations of breast cancer molecular subtypes for Han Chinese population
Source: J Transl Med. 2012 Sep 19;10(Suppl 1):S10. doi: 10.1186/1479-5876-10-S1-S10 (PMC3445863; doi:10.1186/1479-5876-10-S1-S10)
Supplement: Additional file 2 — Supplementary Table S2 Supplementary Table S2 contained pairwise comparisons between 3 intrinsic gene lists for the assignment of the samples. [file 1479-5876-10-S1-S10-S2.pdf]

(a) with gene-centring

	Hu 306						
	Basal	Her2-enriched	Luminal A	Luminal B	Normal breast-like	Unclassified	Total
Sørlie 500							
Basal	37	0	0	0	0	0	37
Her2-enriched	4	14	1	0	2	0	21
Luminal A	0	1	49	19	0	0	69
Luminal B	0	14	1	9	0	0	24
Normal breast-like	0	0	5	0	6	0	11
Unclassified	0	0	2	4	0	1	7
Total	41	29	58	32	8	1	169

	PAM50						
	Basal	Her2-enriched	Luminal A	Luminal B	Normal breast-like	Unclassified	Total
Sørlie 500							
Basal	37	0	0	0	0	0	37
Her2-enriched	4	14	2	0	1	0	21
Luminal A	0	0	44	25	0	0	69
Luminal B	0	15	1	8	0	0	24
Normal breast-like	0	0	6	0	5	0	11
Unclassified	0	1	3	3	0	0	7
Total	41	30	56	36	6	0	169

	PAM50						
	Basal	Her2-enriched	Luminal A	Luminal B	Normal breast-like	Unclassified	Total
Hu 306							
Basal	39	2	0	0	0	0	41
Her2-enriched	1	25	1	2	0	0	29
Luminal A	0	0	52	6	0	0	58
Luminal B	0	3	1	28	0	0	32
Normal breast-like	1	0	1	0	6	0	8
Unclassified	0	0	1	0	0	0	1
Total	41	30	56	36	6	0	169

(b) with DWD adjustment

	Hu 306						
	Basal	Her2-enriched	Luminal A	Luminal B	Normal breast-like	Unclassified	Total
Sørlie 500							
Basal	40	4	0	0	0	0	44
Her2-enriched	1	8	1	0	3	0	13
Luminal A	0	0	47	23	0	0	70
Luminal B	0	12	0	11	0	0	23
Normal breast-like	0	0	9	0	6	0	15
Unclassified	0	1	0	1	1	1	4
Total	41	25	57	35	10	1	169

	PAM50						
	Basal	Her2-enriched	Luminal A	Luminal B	Normal breast-like	Unclassified	Total
Sørlie 500							
Basal	41	3	0	0	0	0	44
Her2-enriched	0	7	1	1	4	0	13
Luminal A	0	1	46	14	9	0	70
Luminal B	0	18	1	4	0	0	23
Normal breast-like	0	0	2	0	13	0	15
Unclassified	0	2	1	0	1	0	4
Total	41	31	51	19	27	0	169

	PAM50						
	Basal	Her2-enriched	Luminal A	Luminal B	Normal breast-like	Unclassified	Total
Hu 306							
Basal	40	1	0	0	0	0	41
Her2-enriched	1	21	1	1	1	0	25
Luminal A	0	1	39	1	16	0	57
Luminal B	0	8	9	17	1	0	35
Normal breast-like	0	0	1	0	9	0	10
Unclassified	0	0	1	0	0	0	1
Total	41	31	51	19	27	0	169
