# Supplementary material for: Prediction consistency and clinical presentations of breast cancer molecular subtypes for Han Chinese population
Source: J Transl Med. 2012 Sep 19;10(Suppl 1):S10. doi: 10.1186/1479-5876-10-S1-S10 (PMC3445863; doi:10.1186/1479-5876-10-S1-S10)
Supplement: Additional file 3 — Supplementary Table S3 Table S3 showed the distributions of molecular subtypes defined by intrinsic genes (Hu 306 and PAM50 with gene-centring and DWD) stratified by subtypes defined by IHC results. [file 1479-5876-10-S1-S10-S3.pdf]

Hu 306 with gene-centring							
IHC subtype	#number	Intrinsic subtype					
		Luminal A	Luminal B	Basal-like	Her2-enriched	Normal breast-like	Unclassified
Luminal A(ER+/HER2-)	73	**55**	16			2	
Luminal B(ER+/HER2+)	23	3	**13**		6		1
Basal-like(ER-/HER2-)	45		2	**38**	3	2	
HER2-enriched(ER-/HER2+)	28		1	3	**20**	4	

Hu 306 with DWD							
IHC subtype	#number	Intrinsic subtype					
		Luminal A	Luminal B	Basal-like	Her2-enriched	Normal breast-like	Unclassified
Luminal A(ER+/HER2-)	73	**53**	18			2	
Luminal B(ER+/HER2+)	23	4	**14**		4		1
Basal-like(ER-/HER2-)	45		2	**38**	3	2	
HER2-enriched(ER-/HER2+)	28		1	3	**18**	6	

PAM50 with gene-centring								
IHC subtype	#number	Intrinsic subtype						
		Luminal A	Luminal B	Basal-like	Her2-enriched	Normal breast-like	Unclassified
Luminal A(ER+/HER2-)	73	**51**	20			2	
Luminal B(ER+/HER2+)	23	3	**14**		6		
Basal-like(ER-/HER2-)	45		2	**37**	4	2	
HER2-enriched(ER-/HER2+)	28	2		4	**20**	2	

PAM50 with DWD							
IHC subtype	#number	Intrinsic subtype					
		Luminal A	Luminal B	Basal-like	Her2-enriched	Normal breast-like	Unclassified
Luminal A(ER+/HER2-)	73	**43**	11			19	
Luminal B(ER+/HER2+)	23	6	**6**		11		
Basal-like(ER-/HER2-)	45	1	1	**37**	4	2	
HER2-enriched(ER-/HER2+)	28	1	1	4	**16**	6	
